# BCL-xL, a Mitochondrial Protein Involved in Successful Aging: From *C. elegans* to Human Centenarians

**DOI:** 10.3390/ijms21020418

**Published:** 2020-01-09

**Authors:** Consuelo Borrás, Cristina Mas-Bargues, Aurora Román-Domínguez, Jorge Sanz-Ros, Lucia Gimeno-Mallench, Marta Inglés, Juan Gambini, José Viña

**Affiliations:** 1 Freshage Research Group, Department of Physiology, Faculty of Medicine, University of Valencia, CIBERFES, INCLIVA, Avenida Blasco Ibañez, 15 46010 Valencia, Spain; cristina.mas@uv.es (C.M.-B.); aurora.roman@ext.uv.es (A.R.-D.); sanzros@alumni.uv.es (J.S.-R.); Lucia.Gimeno@uv.es (L.G.-M.); juan.gambini@uv.es (J.G.); jose.vina@uv.es (J.V.); 2 Freshage Research Group, Department of Physiotherapy, Faculty of Physiotherapy, University of Valencia, CIBERFES, INCLIVA, Avenida Blasco Ibañez, 15 46010 Valencia, Spain; marta.ingles@uv.es

**Keywords:** healthy aging, apoptosis, autophagy, senescence, longevity, mitochondria

## Abstract

B-Cell Lymphoma-extra-large (BCL-xL) is involved in longevity and successful aging, which indicates a role for BCL-xL in cell survival pathway regulation. Beyond its well described role as an inhibitor of apoptosis by preventing cytochrome c release, BCL-xL has also been related, indirectly, to autophagy and senescence pathways. Although in these latter cases, BCL-xL has dual roles, either activating or inhibiting, depending on the cell type and the specific conditions. Taken together, all these findings suggest a precise mechanism of action for BCL-xL, able to regulate the crosstalk between apoptosis, autophagy, and senescence, thus promoting cell survival or cell death. All three pathways can be both beneficial or detrimental depending on the circumstances. Thus, targeting BCL-xL would in turn be a “double-edge sword” and therefore, additional studies are needed to better comprehend this dual and apparently contradictory role of BCL-XL in longevity.

## 1. Introduction to BCL-xL

### 1.1. The BCL-2 Protein Family

BCL-2, the founding member of the BCL-2 family of proteins, was first identified while studying the chromosomal translocation t(14;18) which is a hallmark of several human follicular lymphomas [1,2]. Nevertheless, BCL-2 function was not elucidated until several years later, when Interleukin 3 (IL-3) dependent mouse cell lines were infected with a retroviral vector to express human BCL-2 protein. In the absence of IL-3, BCL-2 promoted cell survival of the infected cells, although they stopped proliferating [3]. Eventually, more proteins having homology sequences with BCL-2 with the capacity to regulate cell death were found. This set of proteins were labeled as the BCL-2 family of proteins.

Currently, it is known that the BCL-2 gene encodes a 26 kD protein consisting of 329 amino acids with a single highly hydrophobic domain at its C-terminus, which enables it to localize mainly in the outer mitochondrial membrane, and, to a lesser extent, in the nuclear envelope and the endoplasmic reticulum. This BCL-2 family of proteins contains all four BCL-2 Homology (BH) domains (BH1, BH2, BH3, and BH4) and a transmembrane (TM) domain. These BH domains facilitate the family members’ interaction with each other, and can result in a pro- or anti-apoptotic function [4,5,6]. More than 600 BCL-2 homologous proteins have been described in metazoan (animal) genomes and are currently available in the BCL2DB database [7]. In order to simplify the study of this large family, members of the BCL-2 family have been classified into three main subgroups by reason of their sequence homology regions [8,9,10]. These subfamilies are (i) Anti-apoptotic proteins, (ii) Pro-apoptotic proteins and (iii) BH3-only proteins.

- The anti-apoptotic proteins possess the four BH regions (BH1–4), and include BCL-2, BCL-xL (BCL2-L1), BCL-W (BCL2-L2), A1 (also known as BFL-1 or BCL-2A1), and MCL-1 (Myeloid cell leukemia 1).
- The pro-apoptotic proteins, which include BAX (BCL-2 associated X protein), BAK (BCL-2 antagonist killer), and BOK/MTD (BCL-2-related ovarian killer/Matador), have three BH domains (BH1–3) and are considered the pore-forming executioners.
- BH3-only proteins generally possess one single BH region, the BH3 motif, and are also sometimes considered as a subcategory of pro-apoptotic proteins owing to their pro-cell death nature. Proteins like BID (BH3-interacting domain death antagonist), BIM/BOD (BCL2L11; BCL2-interacting mediator of cell death), BAD (BCL2 antagonist of cell death), PUMA/BBC3 (p53 up-regulated modulator of apoptosis), NOXA (phorbol-12-myristate-13-acetate-induced protein 1), BIK/BLK/NBK (BCL-2 interacting killer), HRK/DP5 and BMF (BCL-2 modifying factor) are included in this subgroup [11].

The role of the BCL-2 family in the regulation of apoptosis is typically described as the anti-apoptotic and pro-apoptotic BH3-only members existing in a state of competitive flux to influence the activation of the pore forming executioners [12,13]. Once the executioners are activated, they form pores in the outer mitochondrial membrane (MOM), triggering outer mitochondrial membrane permeability (MOMP), and subsequently the cell undergoes apoptosis [14,15,16].

### 1.2. BCL-xL

The human BCL2L1 or BCL-X gene is located in the chromosome 20 (20q11.21). Its DNA contains 58,394 bps (genomic size) and its RNA 2575 bps. The BCL-X gene promoter contains consensus motifs for three transcription factor families, STATs (Signal Transducer and Activator of Transcription), Rel/NF-kB (Rel/Nuclear Factor-κB) and Ets (E26 transformation specific sequence), that have been demonstrated to play an important role in the regulation of the *BCL2L1* gene expression [17].

Alternative splicing of the *BCL-X* gene in human cells results in two major mRNA isoforms: the short isoform *BCL-xS* (591 bp) that has three exons, and the large isoform *BCL-xL* (780 bp) that has four exons. Many cis-regulatory elements and trans-acting factors exert combinatorial control of *BCL-X* splicing. Most known regulators, including Sam68, ASF/SF2 (Alternative Splicing Factor 1 / pre-mRNA-Splicing Factor 2, hnRNPA1 (Heterogenous Nuclear Rinonucleoprotein A1), SRp30c (splicing factor arginine/serine-rich 9 protein), and RBM25 (RNA Binding Motif Protein 25), are able to alter *BCL-X* alternative splicing in vitro or when they are overexpressed in cell cultures [18]. 

#### 1.2.1. BCL-xL Protein Structure

**Figure 1:**
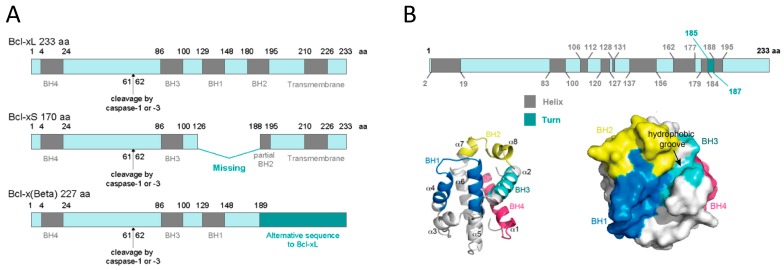
BCL-xL protein structure. (**A**) BCL-xL, BCL-xS and BCL-xβ isoforms. This representation shows the presence of the BH domains as well as the cleavage site by caspase-1 or -3. (**B**) BCL-xL primary, secondary, and tertiary structures. The primary structure shows the linear position of helices and turns; the secondary structure represents the 3D position of the eight helices and the four BH domains; and the tertiary structure reveals the hydrophobic groove (adapted from Lee and Fairlie, IJMS 2019 [24]).

At the protein level, three different transcript variants, which encode distinct isoforms, have been reported. The longer isoform BCL-xL (233 aa) acts as an apoptosis inhibitor and the shorter isoform, BCL-xS (170 aa) acts as an apoptosis activator. The third one, BCL-xβ (227 amino acids) differs from the longer and the shorter isoforms by a modification of the last 45 amino acids, however no specific function has yet been related to this isoform [19]. Figure 1A depicts all three BCL-X isoforms.

Human BCL-xL protein structure is formed by a total of eight α-helices, two of which (α5 and α6) have a central location and are disposed in a parallel fashion [20,21]. These central helices contain predominantly hydrophobic residues and are flanked by α3 and α4 on one side and by α1, α2, and α8 on the other side. Helices α1 and α2 are connected by a flexible 60-residue loop, which is characteristic of the BCL-xL proteins. It is indispensable for translocation to the nucleus [22] and is the main site for post-translational modifications (phosphorylation, deamidation, and cleavage), which have been shown to be efficient ways to regulate the anti-apoptotic function of BCL-xL [23]. In the context of the three-dimensional structure of BCL-xL, the BH domains make essential contributions to its tertiary structure: the BH1 and BH2 domains encompass turn regions linking two helices, α4 to α5 (in the case of BH1) and α7 to α8 (in the case of BH2). The BH3 domain is located entirely on α2 whilst the BH4 domain is located on α1 and makes a number of stabilizing hydrophobic contacts with α2, α5, and α6. Recent findings have proven a major structural feature on BCL-xL protein that is a large hydrophobic groove involving the BH1–BH3 domains. This hydrophobic groove represents the region of greatest difference between the pro-survival proteins. In BCL-xL, α3 and α4 are almost parallel and are relatively tightly packed resulting in a more closed groove. Mutagenesis studies confirm that this cleft could be the site of interaction with pro-apoptotic proteins [24]. Figure 1B shows BCL-xL primary, secondary, and tertiary structures.

BCL-xL has dual mechanisms to regulate apoptosis. First, the hydrophobic groove binds to the α-helical BH3 domain of the pro-apoptotic regulators, inhibiting apoptosis. Second, a site distal to the hydrophobic groove binds to cytosolic p53, inhibiting p53-dependent activation of BAX/BAK, and thus, apoptosis [25,26,27]. Consistent with the postulated multiple modes of action [28,29,30], BCL-xL exists in several conformations, both soluble and in the membrane. Generally, the transition between soluble and membrane conformations can follow two scenarios: anchoring of a relatively unperturbed protein to the lipid bilayer or a complete refolding and bilayer insertion of the protein (e.g., in bacterial toxins). A recent study conducted by Vasquez-Montes et al. [31], demonstrated that the core hydrophobic helix α6 of BCL-xL inserts into the mitochondrial bilayer without adopting a transmembrane orientation. Instead, this insertion disrupts the packing of BCL-xL and releases the regulatory N-terminal BH4 domain (α1) from the rest of the protein structure [31]. 

At the post-translational level, BCL-xL can be phosphorylated, deamidated, and ubiquitinated. Phosphorylation of BCL-xL at Ser-62 and Ser-49, have been well characterized and can alter BCL-xL intracellular localization and its loop conformation [32], which regulates the molecular association with other proteins such as BH3-only proteins and p53 [23]. Interestingly, BCL-xL proteins undergo dynamic phosphorylation/dephosphorylation on Ser-49 and Ser-62 residues during mitosis are important in the maintenance of chromosome integrity in normal cells [33]. The function of BCL-xL is regulated by several kinases, including PLK3 [34], JNK [35], CDK2 [36], and MST1 [37]. Deamidation [38] or ubiquitination [39] of BCL-xL are regulated processes that target it for degradation, and can determine susceptibility to DNA-damaging agents and other death stimuli. 

#### 1.2.2. BCL-xL Protein Localization

**Figure 2:**
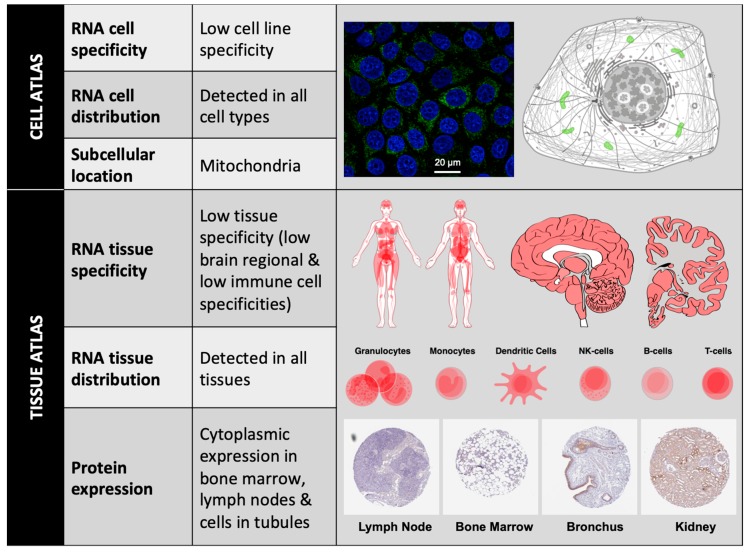
BCL-xL localization. BCL-xL mRNA expression levels can be detected in different cell lines and cell types; BCL-xL protein subcellular location is mainly restricted to mitochondria (Image: MCF7 cells stained with CPTP-BCL2L1-2 antibody obtained from NCI-CPTAC). Consensus data for BCL-xL RNA expression in 55 human tissue types and six blood cell types reveals low tissue specificity. BCL-xL immunohistochemistry in four different tissues stained with HPA035734 antibody (obtained from Atlas Antibodies Sigma-Aldrich). All figures have been obtained from The Human Protein Atlas.

According to The Human Protein Atlas (https://www.proteinatlas.org), at the cellular level, BCL-xL has low RNA cell specificity, as it can be detected in several cell lines such as Caco-2 (colon cancer), MCF7 (mammary gland cancer), HeLa (breast cancer), A549 (lung cancer), etc. In all these cell lines, intracellular localization of BCL-xL protein is mainly in the mitochondria. At the tissue level, BCL-xL has low RNA tissue specificity but the consensus Normalized eXpression (NX) dataset, created as a combination from the three transcriptomics datasets (HPA, GTEx, and FANTOM5), depicts a slight overexpression in bone marrow and thymus. BCL-xL protein levels can be detected in low-medium score in a few tissues, including bone marrow, lymph node, brain, and cells in tubules (Figure 2).

## 2. BCL-xL Role in Mitochondrial Apoptosis

Several models have been proposed to explain how BCL-2 family proteins regulate outer mitochondrial membrane integrity through BAX and BAK:

- The “neutralization model” stipulates that BAX and BAK activation is spontaneous and consequently, neutralization of all anti-apoptotic BCL-2 proteins by BH3-only proteins is sufficient to induce BAX and BAK oligomerization and outer mitochondrial membrane permeability (MOMP) [40,41,42].
- The “direct activation model” posits that BH3-only proteins are divided into “de-repressors/sensitizers” that bind and inhibit anti-apoptotic proteins (e.g., BAD), and “direct-activators” (e.g., BID, BIM) that can also transiently interact with and activate the effectors BAX and BAK [43,44,45].
- The “embedded together” model proposes that anti-apoptotic proteins act as dominant negative regulators of BAX and BAK by binding to direct activators and effectors in membranes, inhibiting both the activation and the oligomerization step [46,47].
- Finally, a more recent study proposed a combination of the previous models and defined two “modes” in which anti-apoptotic proteins prevent apoptosis: by sequestering BH3-only direct activator proteins (MODE 1) or the active effectors BAX and BAK themselves (MODE 2) [48].

All models may be correct and are certainly non-exclusive, and BCL-xL has been reported to play a role in all proposed models. Thus, BCL-xL prevents BAX/BAK oligomerization in the MOM to form a pore for cytochrome c release from mitochondria. In the absence of BCL-xL, cytochrome c exits to the cytosol, and together with Apaf-1 and caspase-9, they form the apoptosome for the dimerization and activation of caspase-9, which, in turn, proteolytically cleaves downstream effector caspases, such as caspase-3, resulting in the activation of these caspases and leading to cell death. Therefore, BCL-xL, preventing cytochrome c release from the mitochondria, prevents apoptosis-mediated cell death.

### BCL-xL Role in Mitochondrial Bioenergetics

The morphology of the mitochondrial population is the result of several interacting dynamical phenomena, including fission, fusion, movement, elimination, and biogenesis. Each of these phenomena is finely controlled by the underlying molecular machinery. Studies in mammals and model organisms have revealed that mitochondrial morphology, dynamics, and function appear to be subjected to regulation by the same proteins that regulate apoptotic cell death [49,50]. Although the biochemical mechanisms are still being investigated, several studies have provided hints regarding the BCL-xL pro-survival function, independently of its canonical role of inhibiting BAX and BAK. Cortical neurons lacking BCL-xL display fragmented mitochondria and restoring BCL-xL levels leads to elongated mitochondria, an enhanced mitochondrial fusion rate, and surprisingly, an even greater increase in the rate of fission. In addition, BCL-xL also increased total mitochondrial biomass (length) in cultured hippocampal neurons and cortical neurons [51,52]. These findings are consistent with the observation that BCL-xL can interact with the mitochondrial fission–fusion machinery [53].

Alavian and colleagues found that, in addition to its outer mitochondrial membrane localization, BCL-xL also localizes in the mitochondrial matrix where it co-immunoprecipitates with the beta subunit of F_1_F_O_ ATP synthase. Exogenously applied, BCL-xL increases F_1_F_O_ ATPase activity, while BCL-xL inhibition reduces the capacity of F_1_F_O_ ATPase-containing sub-mitochondrial vesicles to sequester H^+^ ions. Thus, BCL-xL increases the efficiency of ATP synthesis by decreasing a proton leak within the F_1_F_O_ ATPase, thus improving cellular metabolism and preventing oxidative stress [54]. 

Moreover, phosphorylation of BCL-xL at Ser-62 by the cyclin B1-CDK1 complex in the mitochondria leads to its dissociation from the beta subunit of the F_1_F_O_ ATP synthase. The subsequent inhibition of ATP synthase activity causes complex I oxidative damage, inner mitochondrial membrane depolarization, and apoptotic death [55]. Supporting this fact, a study performed in yeast suggested that, ectopic expression of mammalian BCL-xL allowed cells to survive longer, and this protective effect of BCL-xL was more prominent in respiratory-competent cells that contained defects in mitochondrial ADP/ATP translocation [56].

## 3. BCL-xL Role in Autophagy

Autophagy is an evolutionarily conserved lysosomal pathway for degrading cytoplasmic proteins, macromolecules, and organelles. Three well defined types of autophagy have been established: macroautophagy, endosomal microautophagy, and chaperone-mediated autophagy. However, the current autophagy literature is often viewed as confusing, due to the association with apparently contradictory roles regarding cell survival and cell death [57].

Apoptosis and autophagy are both tightly regulated biological processes that play a central role in tissue homeostasis, development, and disease. The antiapoptotic protein BCL-xL, interacts with the evolutionarily conserved autophagy protein, Beclin-1 [58]. BCL-xL binds and inhibits Beclin-1 and this interaction involves the BH3 domain within Beclin-1 (residues 114–123) [59]. To directly test the impact of the endogenous BCL-xL on autophagy in the absence of apoptosis, Lindqvist and colleagues inhibited their activity in cells lacking the essential cell death mediators BAX and BAK. 

In the absence of BAX and BAK; BCL-xL had no detectable effect on autophagy or cell death in myeloid or fibroblast cell lines. On the other hand, when BAX and BAK were present, inhibition of BCL-xL stimulated autophagy, but this correlated with increased cell death. These results demonstrate that BCL-xL does not directly inhibit components of the autophagic pathway but instead affects autophagy indirectly, owing to the inhibition of Bax and Bak [60,61]. For instance, BCL-xL inhibits both autophagy and apoptosis through interacting respectively with Beclin-1 and BAX/BAK using their BH3-binding pockets [62]. BCL-xL binds to Beclin-1 and thus prevents the interaction between Beclin-1 and the class III PI3K complex (PI3KC3) to inhibit autophagy [63]. Previous studies showed that BCL-xL phosphorylation may be a regulatory switch between autophagy and apoptosis [64,65]. In fact, JNK-mediated phosphorylation mediates the disruption of Beclin-1/BCL-xL and BAX-BAK/BCL-xL interaction, thus triggering autophagy [66,67,68,69].

## 4. BCL-xL Dual Role in Senescence

Like apoptosis, senescence is a cellular response to different stresses characterized by a permanent and irreversible state of cell cycle arrest. Although the determinants by which a cell undergoes senescence or apoptosis are still unclear, both processes seem to be exclusive in physiological conditions. Despite that, a large amount of evidence point to a crosstalk between apoptosis and cellular senescence, in addition to autophagy [70]. 

One of the first studies involving BCL-xL in senescent cells was performed in megakaryocytes derived from CD34+ progenitor cells and UT4 cells, with the aim of clarifying BCL-xL function in the maturation process of different hematopoietic cell lineages. BCL-xL expression was up-regulated during megakaryocyte differentiation [71], and subsequently, senescence was drastically reduced. We also reported that both MEFs and primary cultured human lymphocytes, transduced with a plasmid encoding BCL-xL, downregulated INK/ARF senescent pathway markers, such as p16^INK4a^, p14^Arf^, p21^Cip^, and senescence-associated β-galactosidase (SA-β-GAL) [72]. Taken together, these results suggest a negative correlation between BCL-xL expression and senescence.

In contrast with this data, there is also evidence suggesting that BCL-xL has a positive correlation with senescence. Likewise, BCL-2, BCL-xL is considered as an oncogene due to its ability to promote cell survival. Given that oncogene overexpression can trigger DNA response to damage in order to induce senescence, we could expect that BCL-xL overexpression could also induce this response [73]. In fact, it has been demonstrated that several cell types, when senescent, exhibit high levels of BCL-xL protein. As an example, several triple-negative breast cancer cell lines that survive and undergo cellular senescence after exposure to oncogenic suppressors showed increased basal levels of BCL-xL. Inhibition of BCL-xL in these cells was shown to change cell fates in response to oncogenic suppressors from senescence to apoptosis [74]. In fact, following DNA damage, BCL-xL has the ability to translocate to the nucleus where it binds to CDK1 during the G2/M cell-cycle checkpoint in human lymphoma cell lines. These arrested cells exhibited typical SA-β-GAL activity and were unable to synthesize new DNA when overexpressing BCL-xL, suggesting its capacity to stabilize senescence [22].

**Figure 3:**
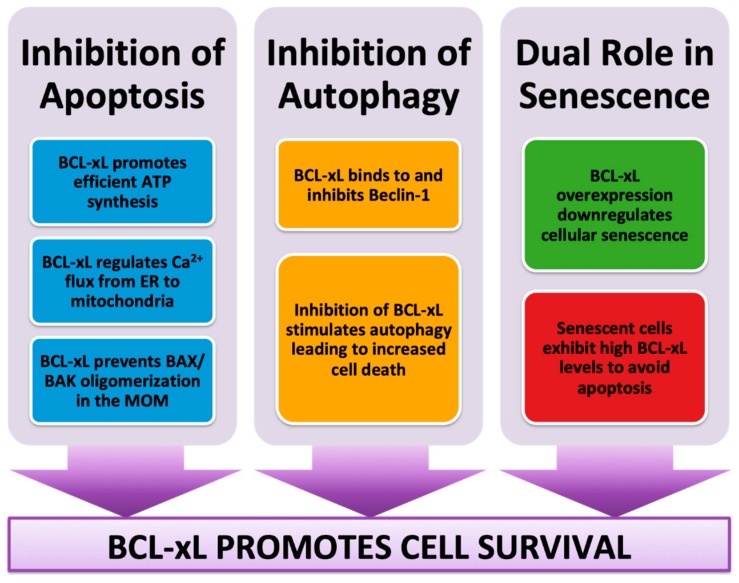
BCL-xL promotes cell survival through apoptosis, autophagy, and senescence regulation.

Overall, it is likely that BCL-xL could both favor and suppress cellular senescence. Presumably, the decision would be dependent on several factors such as the cell type and status (non-tumor or tumor cells), the type and level of stress to which the cell is exposed, and the influence of the surrounding cells and tissues. Nonetheless, further studies are needed towards the understanding of BCL-xL role in cellular senescence in order to elucidate this dual behavior. Summarizing, the main role of BCL-xL is to promote cell survival, either by inhibiting apoptosis or autophagy (or both). Furthermore, it has been described that a certain level of senescence is necessary for successful aging, such as in reprogramming [75], thus high levels of BCL-xL can be beneficial to maintain senescent cells during aging (Figure 3). 

### 4.1. BCL-xL Role in Successful Aging

We have studied centenarians, as an example of successful aging, and have found that they overexpress BCL-xL [72]. By performing functional transcriptomic analysis of peripheral blood mononuclear cells (PMBC), we compared the expression patterns of 28.869 human genes in centenarians, septuagenarians, and young people. Results showed that the mRNA expression pattern of centenarians was similar to the one of young people, and completely different from that of septuagenarians. In particular, sub-network analysis of the 1.721 mRNAs that were found to be statistically different between the three populations, converged on the following six genes: interferon-γ (IFNG), T-cell receptor (TCR), tumor necrosis factor (TNF), SP1 transcription factor, transforming growth factor-β1 (TGFβ1), and cytokine IL-32. 

Likewise, those six genes were related to BCL-xL, Fas, and Fas ligand (FasL), all of them known to be involved in the control of cell death regulation [72]. However, where BCL-xL is an antiapoptotic protein, Fas and FasL are proapoptotic. This could be considered a paradox, as centenarians overexpress anti and proapoptotic proteins at the same time, but there is an explanation: BCL-xL is involved in the inhibition of the intrinsic pathway of apoptosis, which is mainly mediated by mitochondria and activated after self-cell stress; however, Fas and Fas-L induce the extrinsic pathway of apoptosis, which means that they force the cell to die after external stress signals [76]. This suggests that centenarians have a better way to control apoptosis when cells are aging (intrinsic apoptosis), but at the same time, damaged cells by external signals are removed more efficiently (extrinsic apoptosis). 

In order to further demonstrate the role of BCL-xL in longevity, we performed longevity curves using *C. elegans* with a gain function of Ced-9, the ortholog for human BCL-xL. Interestingly, animals overexpressing Ced-9 showed a significant increase in both the mean and the maximum survival time [72]. Although aging is a multifactorial process, these studies suggest that BCL-xL function is relevant in aging and may be one of the factors that contributes to exceptional longevity. 

Moreover, it has been shown that BCL-xL regulates presynaptic plasticity and confers neuroprotection. While other members of the BCL-2 family of proteins substantially decline during development [77], BCL-xL expression is maintained throughout life in the adult brain [78]. This is consistent with the fact that BCL-xL exerts a critical function in mature, long lived neurons. For example, in hippocampal neurons, overexpression of BCL-xL markedly changed synaptic morphology [53]. In the squid giant presynaptic terminal, recombinant BCL-xL protein enhances the rate of recovery of neurotransmission following intense synaptic activity. BCL-xL-regulated recovery depends on stimulation, calmodulin, and BCL-xL translocation to clatherin-coated pits. 

At synaptic vesicles, BCL-xL forms protein–protein interactions with the dynamin-like GTPase Drp1. Depletion of Drp1 or disruption of its interaction with BCL-xL slows down endocytosis and produces aberrantly shaped vesicle membranes [79]. Thus, the underlying molecular details of how BCL-xL alters neuronal synapses may stem from its direct effects on mitochondrial protein complexes as well as on lipids of the inner mitochondrial membrane. These findings could potentially explain the ability of BCL-xL to increase synaptic transmission within minutes when acutely injected into the presynaptic terminus, therefore acting independently of longer-term cell remodeling effects [80].

### 4.2. BCL-xL, a Senolytic Paradox?

It is well known that senescent cells tend to accumulate with age in multiple organisms provoking deleterious effects in neighboring cells and tissues, mainly due to senescence-associated secretory phenotype (SASP) [81]. In fact, cell senescence is considered one of the hallmarks of human aging [82]. In correlation with this, growing experimental evidence has recently demonstrated that the removal of senescent cells prolongs life span and enhances health [83]. Senescent cell clearance was first induced by a drug-activable “suicide” gene in transgenic mice with accelerated aging, resulting in an improvement in age-related phenotypes [84]. Since then, many drugs targeting anti-apoptotic and pro-survival factors have been identified to induce apoptosis in senescent cells in vitro and in vivo [85]. The molecules capable of selectively inducing death in senescent cells are named senolytics [86]. 

In order to identify potential senolytic targets, several anti-apoptotic and pro-survival factors that are upregulated in senescent cells were selected, with BCL-xL among them. Interfering expression of BCL-xL with siRNAs resulted in a reduced viability and survival of senescent but not proliferating human abdominal subcutaneous preadipocytes [87]. Moreover, when administered to old mice, the senolytic drugs Dastainib (D) and Quercetin (Q) were able to delay age-related symptoms and pathologies accompanied with reduced levels of BCL-xL [87]. In the same direction, it has been identified a new senolytic agent, Navitoclax (ABT-263), which induced apoptosis of senescent cells by inhibition of BCL-W and BCL-xL [88,89,90]. 

Similar results were obtained when using the small molecule ABT-737 in human and mice fibroblasts with induced senescence by inhibition of BCL-W and BCL-xL [91]. However, we must not forget that cellular senescence is a response to damage that involves inflammation and extracellular matrix remodeling and that resolves with the phagocytic elimination of the senescent cells. Cellular senescence plays an active and positive role during tissue regeneration, and the full benefits of senescence are achieved when the process includes the clearance of the senescent cells, thereby restoring the pre-damage status of the tissue [92]. Centenarians have presumably higher numbers of senescent cells as well as increased levels of BCL-xL, thus their clearance systems are working properly, offering the utmost benefits of cellular senescence for tissue damage repair. 

Contrarily, we previously described that overexpression of BCL-xL decreases premature senescence in mouse embryo fibroblasts and in human isolated lymphocytes [72]. After four passages in culture, BCL-xL overexpressing cells exhibited lower levels of p16^Ink4a^, p14^Arf^, and p21^Cip^, all known to inhibit the cell cycle. Moreover, as discussed before, BCL-xL overexpression is found in individuals with extreme longevity. 

In contrast, nematodes bearing a gain of function mutation in the BCL-xL ortholog ced-9 showed increased longevity, suggesting that BL-xL is involved in healthy aging [72]. Thus, we get to the following paradox by which BCL-xL is elevated in some senescent cells and its inhibition results in several beneficial effects for the individual, whereas BCL-xL overexpression reduces senescence markers in other cells. Similarly, there is also the contradiction that not only does overexpression of BCL-xL seem to play a role in longevity and life span, but also the interference of its expression in senescent cells has been linked to a wide range of beneficial effects.

## 5. Conclusions

In conclusion, BCL-xL is a complex protein involved in the three main defense pathways of cells: apoptosis, autophagy, and senescence. All three are interconnected and can be both beneficial and detrimental depending on the circumstances. BCL-xL localizes in the mitochondria, where is able to protect them by promoting efficient ATP synthesis and regulating Ca^2+^ flux from the endoplasmic reticulum, contributing to the energy balance, which is very important for healthy aging. Under healthy conditions, BCL-xL levels maintain cell survival directly by inhibiting apoptosis, or indirectly by repressing autophagy. Successful aging, such as the case of centenarians, is associated with high levels of BCL-xL. However, in ordinary aging, senescent cells may accumulate due to an inefficient immune system, and in this case, BCL-xL should be well monitored. Thus, targeting BCL-xL would, in turn, be a “double-edge sword” and therefore, additional studies are needed to better comprehend these dual and apparently contradictory roles of BCL-XL in longevity.
